# Emergence of SARS-CoV-2 Variant B.1.575.2, Containing the E484K Mutation in the Spike Protein, in Pamplona, Spain, May to June 2021

**DOI:** 10.1128/JCM.01736-21

**Published:** 2021-11-18

**Authors:** Camino Trobajo-Sanmartín, Ana Miqueleiz, María Eugenia Portillo, Miguel Fernández-Huerta, Ana Navascués, Pilar Sola Sara, Paula López Moreno, Gonzalo R. Ordoñez, Jesús Castilla, Carmen Ezpeleta

**Affiliations:** a Departament of Clinical Microbiology, Complejo Hospitalario de Navarra, Pamplona, Spain; b Instituto de Investigación Sanitaria de Navarra, Pamplona, Spain; c Servicio de Urgencias Extrahospitalarias, Pamplona, Spain; d Centro de Secuenciación NASERTIC, Pamplona, Spain; e Instituto de Salud Pública de Navarra, Pamplona, Spain; f CIBER Epidemiología y Salud Pública, Madrid, Spain; Cepheid

**Keywords:** SARS-CoV-2, E484K mutation, lineage B.1.575, variant, sequencing

## Abstract

With the emergence of new severe acute respiratory syndrome coronavirus 2 (SARS-CoV-2) variants and the acquisition of novel mutations in existing lineages, the need to implement methods capable of monitoring viral dynamics arises. We report the emergence and spread of a new SARS-CoV-2 variant within the B.1.575 lineage, containing the E484K mutation in the spike protein (named B.1.575.2), in a region in northern Spain in May and June 2021. SARS-CoV-2-positive samples with cycle threshold values of ≤30 were selected to screen for presumptive variants using the TaqPath coronavirus disease 2019 (COVID-19) reverse transcription (RT)-PCR kit and the TaqMan SARS-CoV-2 mutation panel. Confirmation of variants was performed by whole-genome sequencing. Of the 200 samples belonging to the B.1.575 lineage, 194 (97%) corresponded to the B.1.575.2 sublineage, which was related to the presence of the E484K mutation. Of 197 cases registered in the Global Initiative on Sharing Avian Influenza Data (GISAID) EpiCoV database as lineage B.1.575.2, 194 (99.5%) were identified in Pamplona, Spain. This report emphasizes the importance of complementing surveillance of SARS-CoV-2 with sequencing for the rapid control of emerging viral variants.

## INTRODUCTION

During the severe acute respiratory syndrome coronavirus 2 (SARS-CoV-2) pandemic, several variants that were catalogued as variants of concern (VOCs) or variants of interest (VOIs) by the European Centre for Disease Prevention and Control have emerged in different countries. As of 23 June 2021, the four important lineages with evident impact on transmissibility, severity, and immunity are lineages B.1.1.7 (Alpha), B.1.351 (Beta), B.1.617.2 (Delta), and P.1 (Gamma) (1–4). Lineages B.1.351 and P.1 are of specific concern because they present the spike mutation E484K, which has been associated with reduced neutralizing activity of antibodies and may be associated with reduced efficacy of vaccines (2, 3, 5, 6). Initially, the B.1.1.7 lineage had mutations N501Y and D614G and the characteristic ΔH69/ΔV70 deletion in the spike protein; in early 2021, however, Public Health England reported the first B.1.1.7 SARS-CoV-2 cases that had acquired the E484K mutation (3, 7). In this regard, concerns about the emergence of new SARS-CoV-2 variants and the acquisition of new mutations in existing lineages, such as the accumulation of mutations in the spike gene in B.1.1.7, have been developing since the onset of the pandemic.

The lineage B.1.575 emerged in the United States, and since its emergence two new sublineages have been identified. The B.1.575.1 sublineage was classified in the Phylogenetic Assignment of Named Global Outbreak (PANGO) lineage system as a Spanish sublineage of B.1.575 with spike mutations P681H, S494P, and T716I, and the B.1.575.2 sublineage, whose main characteristic is the presence of the E484K spike mutation, also originated in Spain (8).

This study identified the emergence and spread of the E484K spike mutation within the SARS-CoV-2 B.1.575.2 lineage, which increased in the circulating virus population in Pamplona, Spain, between May and June 2021. Additionally, we share our experience with the prospective surveillance of novel SARS-CoV-2 variants by implementing a two-step laboratory strategy, i.e., reverse transcription quantitative real-time PCR (RT-qPCR) screening and whole-genome sequencing.

## MATERIALS AND METHODS

The Microbiology Department of the Complejo Hospitalario de Navarra, which is located in Pamplona, the capital city of Navarra, Spain (with approximately 650,000 inhabitants), is the reference laboratory of the public health system for SARS-CoV-2. Upper respiratory specimens for SARS-CoV-2 detection are routinely collected at hospitals and primary care centers and processed by commercial RT-qPCR methods. Since the end of 2020, when variant B.1.1.7 became predominant in the United Kingdom, prospective sample-based surveillance has been conducted in our community to identify novel emerging SARS-CoV-2 variants. A two-step laboratory procedure includes all positive SARS-CoV-2 samples from hospital patients and community settings with a cycle threshold (*C_T_*) value of ≤30. Occasionally, targeted samples are also included according to epidemiological criteria.

Screening of presumptive SARS-CoV-2 variants carrying the ΔH69/ΔV70 deletion was performed using the TaqPath coronavirus disease 2019 (COVID-19) RT-PCR kit (Thermo Fisher Scientific, USA), following the manufacturer’s instructions. Then, all samples of non-B.1.1.7 variants were subjected to a second RT-qPCR assay with the TaqMan SARS-CoV-2 mutation panel (Thermo Fisher Scientific). At that time, we customized the TaqMan assay to detect SARS-CoV-2 spike protein with the N501Y, E484K, K417N, and K417T mutations. All samples were sequenced.

Whole-genome sequencing was performed using the COVIDSeq test (Illumina Inc., USA) on the Illumina NovaSeq 6000 system located in the public company NASERTIC, following the manufacturer’s instructions. The viral lineage classifications were performed with the Global Initiative on Sharing Avian Influenza Data (GISAID) EpiCoV database (https://www.gisaid.org), Nextstrain (https://nextstrain.org), and the PANGO lineage system (https://cov-lineages.org) (9–11).

### Data availability.

All genomes generated in this work were deposited in the GISAID EpiCoV database (http://gisaid.org) under accession numbers EPI_ISL_2510533, EPI_ISL_2510585 to EPI_ISL_2510589, EPI_ISL_2510592, EPI_ISL_2516620, EPI_ISL_2516686 to EPI_ISL_2516689, EPI_ISL_2516691, EPI_ISL_2516698 to EPI_ISL_2516704, EPI_ISL_2516709, EPI_ISL_2516710 to EPI_ISL_2516715, EPI_ISL_2516717 to EPI_ISL_2516721, EPI_ISL_2516723, EPI_ISL_2516724, EPI_ISL_2516726 to EPI_ISL_2516728, EPI_ISL_2516731, EPI_ISL_2516732, EPI_ISL_2516734 to EPI_ISL_2516749, EPI_ISL_2516753, EPI_ISL_2516754, EPI_ISL_2516756, EPI_ISL_2516757, EPI_ISL_2516759 to EPI_ISL_2516761, EPI_ISL_2516855 to EPI_ISL_2516857, EPI_ISL_2516861 to EPI_ISL_2516877, EPI_ISL_2516879, EPI_ISL_2516881, EPI_ISL_2516934, EPI_ISL_2516937 to EPI_ISL_2516940, EPI_ISL_2934576 to EPI_ISL_2934590, EPI_ISL_2934594, EPI_ISL_2934597, EPI_ISL_2934599 to EPI_ISL_2934603, EPI_ISL_2934617 to EPI_ISL_2934623, EPI_ISL_2934625, EPI_ISL_2934626, EPI_ISL_2934629 to EPI_ISL_2934635, EPI_ISL_2934638 to EPI_ISL_2934640, EPI_ISL_2934642, EPI_ISL_2934645 to EPI_ISL_2934651, EPI_ISL_2934656 to EPI_ISL_2934659, EPI_ISL_2934661, EPI_ISL_2934662, EPI_ISL_2934665 to EPI_ISL_2934669, EPI_ISL_2934672, EPI_ISL_2934673, EPI_ISL_2934676 to EPI_ISL_2934681, EPI_ISL_2934684 to EPI_ISL_2934686, EPI_ISL_2934699, EPI_ISL_2934703 to EPI_ISL_2934722, EPI_ISL_2934728 to EPI_ISL_2934730, EPI_ISL_2934844, EPI_ISL_2934846 to EPI_ISL_2934848, EPI_ISL_2934852, EPI_ISL_2934853, EPI_ISL_2934897, EPI_ISL_2934905, EPI_ISL_2934906, EPI_ISL_2934910, and EPI_ISL_2934917 (B.1.575.2), EPI_ISL_1392993 and EPI_ISL_1393347 (B.1.575.1), and EPI_ISL_1622537, EPI_ISL_1622538, EPI_ISL_1622541, and EPI_ISL_1622850 (B.1.575).

## RESULTS

A total of 4,728 SARS-COV-2 genomes had been sequenced in Navarra by 1 August 2021. Our sequencing analysis identified 200 samples (4.2% of the total) related to the B.1.575 lineage, i.e., 4 (2%) B.1.575, 2 (1%) B.1.575.1, and 194 (97%) B.1.575.2. Among the common substitutions present in these lineages, four occurred in the spike protein (S494P, D614G, P681H, and T716I) (12). All samples showed as gene S positive (not carrying the ΔH69/ΔV70 deletion) in the TaqPath assay. In the TaqMan assay, all samples identified by sequencing as B.1.575.2 showed the E484K mutation.

**FIG 1:**
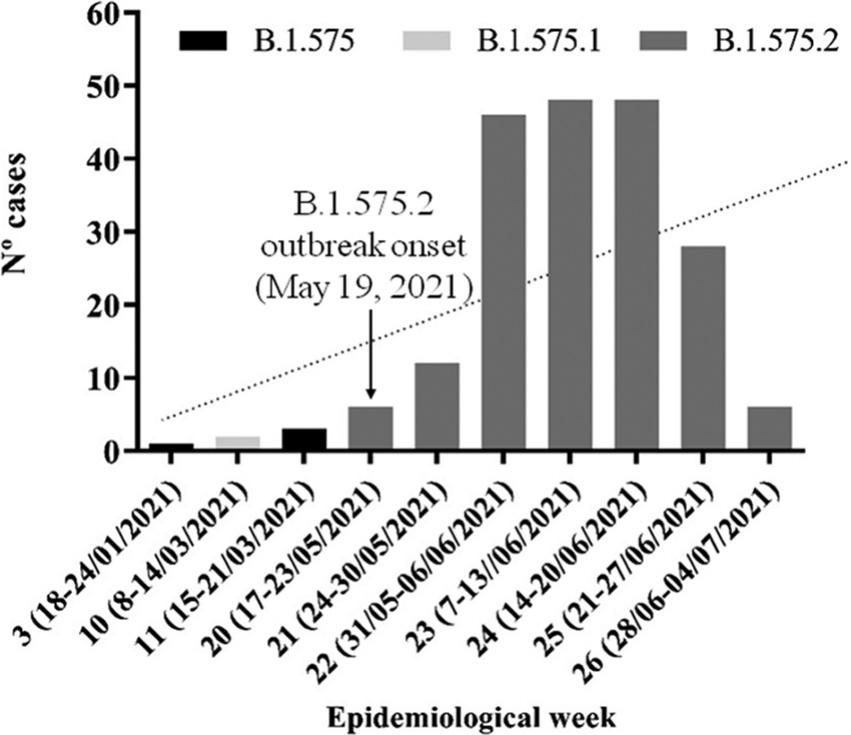
Timeline of the emergence of the SARS-CoV-2 B.1.575, B1.575.1, and B.1.575.2 lineages in Pamplona, Spain, between January and June 2021.

The first case with the B.1.575 lineage to be identified in Pamplona dates back to 20 January 2021; after that date, no other case was identified until 15 March 2021, when three isolates showing mutations common to the B.1.575 lineage were recorded. Between week 20 and week 26 of 2021, we identified 194 cases with lineage B.1.575, which had acquired another S mutation, E484K, classified in the GISAID EpiCoV and Pangolin databases as representing sublineage B.1.575.2. The first case with the B.1.575.2 lineage was identified in a sample isolated on 19 May 2021 (week 20 of 2021); the number of cases grew to 48 cases in weeks 23 and 24 and declined suddenly at the end of June due to the emergence of the Delta (B.1.617.2) variant (Fig. 1). This variant is more infectious and is leading to increased transmissibility, compared with other variants. Delta is currently the predominant variant in Spain. The beginning of the outbreak was detected in a car repair shop located in a district of Pamplona. These cases could be related to another more significant outbreak of variant B.1.575.2 that was found in a mosque. Since those first cases, the variant has spread throughout Pamplona and its surroundings without affecting the rest of Navarra. The median age of the patients was 33 ± 17 years, with 43.8% women and 56.2% men and approximately 50% of Arabic origin. Eighty-two patients (42.3%) acquired the infection in a domiciliary setting, the most common cause. Only 14 (7.2%) acquired the infection at the workplace. One hundred fifty-six patients (80.4%) showed symptoms, but only 4 patients (2.1%) were admitted to hospitals, and no one suffered a severe form of the disease. Twelve patients (6.2%) were fully vaccinated for COVID-19, 6 patients (3.1%) with two doses of Comirnaty (BNT162b2 mRNA; BioNTech-Pfizer, Mainz, Germany) and 6 patients with one dose of Janssen vaccine (Ad26.COV2-S; Janssen-Cilag International NV, Beerse, Belgium). Twenty-four patients (12.4%) were partially vaccinated: 11 (5.7%) with Comirnaty, 4 (2.1%) with Spikevax (mRNA-1273; Moderna, Cambridge, MA, USA), and 9 (4.6%) with Vaxzevria (ChAdOx1 nCoV-19; Oxford-AstraZeneca, Cambridge, UK) (Table 1). Among fully vaccinated patients, most (9/12 patients [75%]) showed symptoms.

**TABLE 1:** Characteristics of the patients with SARS-CoV-2 B.1.575.2 lineage included in the study

Parameter	No. (%)
Age group	
≤19 yr	48 (24.7)
20–39 yr	72 (37.1)
40–59 yr	67 (34.5)
≥60 yr	7 (3.6)
Sex	
Male	109 (56.2)
Female	85 (43.8)
Origin	
Arabic	98 (50.5)
Non-Arabic	96 (49.5)
Contact setting	
Domiciliary	82 (42.3)
Nondomiciliary	112 (57.7)
Symptomatic COVID-19	
Yes	156 (80.4)
No	38 (19.6)
Admission to hospital	
Yes	4 (2.1)
No	190 (97.9)
Covid-19 vaccination status	
Unvaccinated	158 (81.4)
Partially vaccinated	24 (12.4)
Fully vaccinated	12 (6.2)
Covid-19 vaccination	
Comirnaty	17 (8.8)
Janssen	6 (3.1)
Spikevax	4 (2.1)
Vaxzevria	9 (4.6)
Total	194 (100)

To determine the distribution of the SARS-CoV-2 B.1.575 lineage, we searched in the GISAID EpiCoV and PANGO lineage databases. From May to July, the lineage and sublineages of B.1.575 have increased exponentially in different countries. The B.1.575 lineage was predominant in the United States (90%), while the B.1.575.1 and B.1.575.2 sublineages dominated in Spain (86% and 92%, respectively).

The B.1.575.2 sublineage was predominant in Navarra, since 99.5% of the cases (194/197 cases) registered in the GISAID EpiCoV database were identified in this region. In contrast, we did not identify any genomes of the B.1.575 or B.1.575.1 lineage carrying the E484K mutation.

## DISCUSSION

In this study, we observed the emergence of lineage B.1.575.2 with the spike E484K mutation, circulating in Pamplona in association with an outbreak. Pamplona is a small city located in the north of Spain, near France, and it could serve as a spread model for other cities in the world. The new lineage displayed a low prevalence (4.10%) among SARS-CoV-2 genomes analyzed between 23 March 2020 and 30 June 2021. Still, it was already dispersed in our city and represented 97% of the B.1.575 sequences detected during that period. The E484K mutation is considered one of the most important substitutions associated with reduced antibody neutralization potency and efficacy of the SARS-CoV-2 vaccine (13–15). The E484K mutation has been identified in SARS-CoV-2 variants considered VOCs, such as B.1.351, P.1, and B.1.1.7+E484K and in VOI variants, such as B.1.525, B.1.620, and B1.621, among others (1–3); therefore, the presence of this mutation should be monitored.

Screening PCR is a useful tool for detecting mutations, mainly because of its rapidity. Future identification with this method, including new mutations characteristic of the lineage, could serve as a rapid method of variant identification. However, whole-genome sequencing remains the gold standard technique for pandemic control.

To our knowledge, this is the first study that describes the emergence of the lineage B.1.575.2. This genetic variant includes a mutation in the spike protein (E484K). This SARS-CoV-2 genetic variant was discovered in Pamplona in association with an outbreak, demonstrating the importance of genetic sequencing, especially for new community outbreaks.

This brief report emphasizes the importance of exhaustive surveillance for circulating variants of SARS-CoV-2, to reduce community transmission, to assess the COVID-19 vaccine effectiveness, and to prevent the emergence of more transmissible variants that could further increase the severity of the epidemic in the country.
